# A Year’s Progress in Medicine1An address delivered before the Alumni Association, University of Buffalo, March 24, 1891.

**Published:** 1891-05

**Authors:** P. M. Wise

**Affiliations:** Superintendent St. Lawrence State Hospital, Ogdensburg, N. Y.


### A YEAR'S PROGRESS IN MEDICINE.[1]

By P. M. WISE, M. D.,

Superintendent St. Lawrence State Hospital, Ogdensburg, N. Y.

ANOTHER wave of time has left its impress upon its eternal sands, since last the members of this Association gathered under the roof of our gracious mother. In the infinite lapse of years, it will be a mere forgotten atom, and even in the span of our own generation, it will, perhaps, be one of many marked with no unusual incident, yet it has been pregnant with hope and despair to some. We meet here year by year, as the speed of time increases with departed and departing youth, and grasp the hands of fellows who, side by side, remain true to the impulse that first led them here. Since we last met as an Association, some of our brethren have gone to their long home. The work of their life has been laid aside, and they have passed to the great majority. May they rest in peace and meet the reward that is held for the faithful!

Others have come to join us, and are looking hopefully in the rose-colored dawn of professional inspirings to a future of progressive success. Alas! my young brother, prepare for disappointment, but in this gladsome hour do not become infected by the pessimism of the white-haired brother by your side. Keep your youth, with its hopefulness, as long as you can, and let its tonic infection do for your client what you will often fail to meet with therapeutics. Cheer with natural exuberance as long as you can, for cheer you must, whether the heart be burdened and the marks of sorrow have effaced the smiling countenance. The smile must always come, the words of hope must find utterance, the presence of the physician must be surrounded by an atmosphere essentially its own, characterized by comfort and hope and cheer.

The year has been prolific in theories and discoveries that will

1. An address delivered before the Alumni Association, University of Buffalo, March 24, 1891.

mark it for generations to come. Perhaps no one occasion of medical advance has been attended with greater popular excitement since the time of Jenner, than the announcement of Koch that a culture of tubercle bacilli after specific preparation would cause a reaction easily recognized in cases of tuberculosis. The diagnostic value of the Koch process is undoubtedly very great. Its curative properties have been over-rated, but so short a time has elapsed since its introduction to the profession, that it is eminently unfair to judge its therapeutic properties. From what we know of it we may feel that it involves a principle which in some form will modify the terrors of a disease that to millions of families has been a veritable scourge. It is a great step in the bacteriological treatment of disease—and lies in the course of the most probable advances in medicine and the most wonderful discoveries. Like the etiology and pathology of diseases the specific poison of which has been found to reside in fibrionic material, so in the cure of such diseases and as a resistance against them, the signs of the times indicate that some modification of the poison will prove a strong factor. In surgery, among other important progress, it has been shown that tissues of the lower animals will unite with human tissues. This opens an immense field for experiment and research. The continual advances in abdominal surgery has not been checked during the past year. In all branches of medicine there has been an activity that has never been equalled in the history of our noble science and the outlook has never been brighter. In the care and treatment of the insane, regarding which I can speak to you with more positiveness, there has been established in this State by statutory law, a principle that I hope will live to the end of time, and that is, that such sick as are legally exempt from crime and are legally incompetent through insanity, shall become the wards of the Commonwealth, where their care and treatment can be established and supervised on broad and human lines and not be left to localities. In this connection it is gratifying to note that this neglected disease in the past is receiving more and more attention as its importance demands. In my own recollection within the past twenty years, it has been my fortune to witness a gradual and marked progress in the knowledge of insanity on the part of the general practitioner as manifested in certificates of insanity. It is gatifying to note also, that our own *alma mater* was among the first colleges in the country to enlarge her curriculum to embrace this as a study. There is within easy reach unusual opportunities for the study of insanity in one of the best hospitals in our country, and, in this respect, it far exceeds the advantages presented by the larger metropolitan schools. Its executive head, who is also an instructor here, is an acknowledged leader in his special work, and no excuse exists for the graduate from this school at the present day who fails to carry with him a good working knowledge of insanity. Brethren of '72 and previous, we may rejoice that what was denied us is assured to our children.

One particular province of the family physician, in close connection with his professional work, is in the line of prevention of nervous diseases and insanity in his capacity as family adviser. I never lose an opportunity of emphasizing this fact and the diversion may be charged to my weakness. The amount of good work that can be done in the education of families, by impressive illustration of the evils of injudicious training of children and daily living and habits as well as the evil results springing from unwise marriages, upon occasions that are only presented to the family physician, can hardly be measured. The word of the doctor outweighs that of the minister or the lawyer in the family council. From the *colica flatulenta* of infancy to the *colica menstrualis* of womanhood; from the præputial to the seminal period and thence to the climateric and to senility, his words of counsel prevail in the serious family conclave and in the closet. How often by a discreet word might he avert calamities that to his practised eye can be foreseen. What more noble function than to point out plainly and practically, not in theory, the avoidance of a road that leads to chorea, epilepsy, insanity and death and, perhaps, worse than death; that strews a life-path, holding possibilities of comparative ease and happiness, with the thorns of sorrow that spring from avoidable causes? O, if some thoughtful physician had but given a word of warning! This is not an infrequent cry. It comes from retrospection. It has been caused by ignorance.

The teaching of physiology in schools is beginning to show some good results in a more enlightened youth. We may look forward to a day when the physician's struggle with ignorance will be lessened; when he can appeal to reason and not to prejudice; when our law makers will have reached a degree of intelligence sufficient to legislate for other than political purposes, and when the public health can be protected from the greatest of all degenerative causes —heredity.

The neurotic diathesis is more difficult to meet than any other of the constitutional tendencies to disease. The influences that operate toward development of nervous disease where there is an existing predisposition are subtle. The so-called moral causes are excitants, and the habits and manner of life are potent factors. The training of youth is an opportunity for preventive medicine that, it seems to me, has never been given its full share of attention, for physical discipline in nerve development plays as important a part as education in the training of the mind. When degeneration has positively asserted itself the danger cannot be averted, but there can be no doubt that the unstable nervous elements can be strengthened by proper physical and mental training. In this field lies an opportunity that should not be withheld and cannot be lost. Some measure of comparison is presented in the undeveloped, or arrested development of nerve organs. Here we have healthy structure, but without growth.

In the neurote we have growth without stability. Now in either case development is the only means at our command,—in one case through stimulation of developed structure, and in the other case through physical renovation a reformation of structure. Functional growth can also be directed by intellectual means.

Among the causes of the increase of insanity,—or rather the increase in the aggregate of insane, for I do not admit that primary occurring cases of insanity are increasing,—it is a very noticeable fact in institutions that much of it is due to the secondary insanities; that is, to insanity that follows some other expression of disease of the nervous system. Thus formerly it was the rule to receive three cases of primary insanity in an acute form from five admissions, while now it may be safely stated that the three are insanities that are the culmination of degenerated nerve structure in its portean forms. In a corresponding ratio the recoveries have decreased. Without entering any other reason for this, it is my only purpose to emphasize the necessity to recognize the increasing tendency to nervous disease other than insanity; and also those expressions of weak, non-resistent organizations, that may luckily for themselves pass their generation without a breakdown, but unluckily for posterity procreate weak and unbalanced organizations that *will* break down and that will help to swell the increasing army of neurotes.

A question that makes an alienist wish for rest, is one that is aimed at him constantly with the expectation that it will be answered concisely in a few short, sharp, crisp words, "Doctor, what is the cause of insanity?"

Alas! that it might be answered without viewing the long catalogue of foibles and weaknesses and vices of the human race: that one might look to some mammoth cause against which could be directed the whitest light of investigation, and the mightiest power of elimination. But the myriads of subtle, creeping, insidious sources of degeneration sapping the foundation of healthy nerve structure, transmitting to progeny its acquired weaknesses in increased measure, and culminaiing in that perversion of the ego, —that is death in life. This the student of causes stands amazed before. How can it be met? This is a question for this and the succeeding generations. Surely it seems to me there is no method short of thwarting the procreation of the "unfit" and fostering the increase and "survival of the fittest." Until the necessity for race preservation actuates legislators, the only amelioration lies in the mission work that can be done by our noble army of health counselors.

It is to be hoped that the vital question of increased accommodations for the healthy growth of our medical school will receive appropriate attention at this meeting of the Association.

Whether far removed from its active life or in the midst of it, we all have an abiding interest in its success. The medical department of this University has a history that we may feel a common pride in, and it is sincerely to be hoped that the great need that now exists for increased and larger accommodations may be met in some satisfactory way. The committee appointed at the last annual meeting will report their progress. What we can do as a body or as individuals in strengthening their hands should be willingly, lovingly done, and although we may not relieve the necessity, we may aid its final accomplishment.

---

Hot water is one of our best remedial agents. A hot bath on going to bed, even in the hot nights of Summer, is a better reliever of insomnia than many drugs.

Inflamed parts will subside under the continual poulticing of real hot water.

Very hot water, as we all know, is a prompt checker of bleeding, and besides, if it is clean, as it should be, it aids us in sterilizing our wound.

A riotous or rotten stomach will nearly always receive gratefully a glass or more of hot water.—*Medical Mirror*.

